# Phenotyping and outcome on contemporary management in a German cohort of patients with peripartum cardiomyopathy

**DOI:** 10.1007/s00395-013-0366-9

**Published:** 2013-06-28

**Authors:** A. Haghikia, E. Podewski, E. Libhaber, S. Labidi, D. Fischer, P. Roentgen, D. Tsikas, J. Jordan, R. Lichtinghagen, C. S. von Kaisenberg, I. Struman, N. Bovy, K. Sliwa, J. Bauersachs, Denise Hilfiker-Kleiner

**Affiliations:** 1 Department of Cardiology and Angiology, Medical School Hannover, Carl-Neuberg-Str. 1, 30625 Hannover, Germany; 2 Department of Medicine, Faculty of Health Sciences, Hatter Cardiovascular Research Institute, University of Cape Town, Cape Town, South Africa; 3 School of Clinical Medicine, University of the Witwatersrand, Johannesburg, South Africa; 4 Department of Clinical Pharmacology, Medical School Hannover, Hannover, Germany; 5 Department of Clinical Chemistry, Medical School Hannover, Hannover, Germany; 6 Department of Gynecology and Prenatal Medicine, Medical School Hannover, Hannover, Germany; 7 Unit of Molecular Biology and Genetic Engineering, GIGA, University of Liège, Liège, Belgium

**Keywords:** Peripartum cardiomyopathy, Registry, Prolactin, MicroRNA, Biomarker

## Abstract

Peripartum cardiomyopathy (PPCM) is a life-threatening heart disease developing towards the end of pregnancy or in the months following delivery in previously healthy women in terms of cardiac disease. Enhanced oxidative stress and the subsequent cleavage of the nursing hormone Prolactin into an anti-angiogenic 16 kDa subfragment emerged as a potential causal factor of the disease. We established a prospective registry with confirmed PPCM present in 115 patients (mean baseline left ventricular ejection fraction, LVEF: 27 ± 9 %). Follow-up data (6 ± 3 months) showed LVEF improvement in 85 % and full recovery in 47 % while 15 % failed to recover with death in 2 % of patients. A positive family history of cardiomyopathy was present in 16.5 %. Pregnancy-associated hypertension was associated with a better outcome while a baseline LVEF ≤ 25 % was associated with a worse outcome. A high recovery rate (96 %) was observed in patients obtaining combination therapy with beta-blocker, angiotensin-converting enzyme (ACE) inhibitors/angiotensin-receptor-blockers (ARBs) and bromocriptine. Increased serum levels of Cathepsin D, the enzyme that generates 16 kDa Prolactin, miR-146a, a direct target of 16 kDa Prolactin, N-terminal-pro-brain-natriuretic peptide (NT-proBNP) and asymmetric dimethylarginine (ADMA) emerged as biomarkers for PPCM. In conclusion, low baseline LVEF is a predictor for poor outcome while pregnancy-induced hypertensive disorders are associated with a better outcome in this European PPCM cohort. The high recovery rate in this collective is associated with a treatment concept using beta-blockers, ACE inhibitors/ARBs and bromocriptine. Increased levels of Cathepsin D activity, miR-146a and ADMA in serum of PPCM patients support the pathophysiological role of 16 kDa Prolactin for PPCM and may be used as a specific diagnostic marker profile.

**Electronic supplementary material:**

The online version of this article (doi:10.1007/s00395-013-0366-9) contains supplementary material, which is available to authorized users.

## Introduction

Peripartum cardiomyopathy (PPCM) is the major cause of pregnancy-induced heart failure and is associated with high morbidity and mortality [16, 19, 21, 24].

The true incidence of PPCM is unknown, as clinical presentation varies. Current estimates ranging from 1:299 (Haiti), 1:1000 (South Africa) to 1:3186 in the USA are primarily based on case series from single centres or retrospective questionnaires [2, 3]. No data exists on the frequency of the disease in Europe whose pathophysiology remains still unclear with multiple factors likely to contribute and to drive progression. Nevertheless, advances have been achieved in understanding some underlying molecular cascades deregulated in PPCM pointing to an important role of an angiogenic imbalance caused by angiostatic and pro-apoptotic 16 kDa Prolactin fragment and the soluble VEGF receptor 1 (sFlt1) which leads to massive endothelial damage and myocardial dysfunction [9, 10, 15]. 16 kDa Prolactin seems to mediate a large part of its anti-angiogenic effects by the induction of microRNA-146a (miR-146a) [9]. In endothelial cells miR-146a inhibits proliferation and promotes apoptosis [9]. Moreover, 16 kDa Prolactin promotes shedding of miR-146a loaded exosomes from endothelial cells that are absorbed by cardiomyocytes where they impair metabolic activity [9], a feature that is further supported by observations showing that endothelial microparticles are increased in acute PPCM [26]. Genetic factors may contribute to the susceptibility to PPCM in patients with positive family history of cardiomyopathy, who typically have a more severe course of disease [14, 25].

In order to gain further insight into the contemporary epidemiology, diagnosis, etiology and management of PPCM patients, we established a prospective registry for PPCM cases between 2004 and 2012 in Germany with patients who were newly diagnosed with PPCM according to the definition proposed in a recent position paper from the Heart Failure Association of the European Society of Cardiology [24]: (1) PPCM is an idiopathic cardiomyopathy presenting with heart failure secondary to left ventricular (LV) systolic dysfunction towards the end of pregnancy or in the months following delivery, where no other cause of heart failure is found; (2) it is a diagnosis of exclusion with a left ventricular ejection fraction (LVEF) nearly always reduced below 45 % but not always associated with LV dilatation. From this first larger prospective German PPCM cohort we believe to gain novel insights on etiology, risk factors, underlying pathophysiology, co-morbidity, prognosis, biomarker profiles and therapeutic concepts of PPCM in Western European societies.

## Methods

### Data collection

Our local Ethics Committee approved this study. Suspected PPCM cases were reported to our registry from University hospitals, tertiary hospitals or cardiologists in private practice. All patients provided a written informed consent, minimal requirement for enrolment was baseline LVEF and close relationship to pregnancy (pregnant or delivery up to 6 months ago); not all patients agreed to provide probes and additional clinical data, a reason why not all other data sets are complete in this registry. Clinical assessments such as onset of symptoms and signs during first presentation, New York Heart Association (NYHA) functional class, ECG, echocardiographic analyses, family history, diseases in pregnancy and mode of delivery were obtained from the patients, the referring physician and by examining the obstetric cards and medical records. Clinical and laboratory assessments were performed in 115 patients with confirmed PPCM at time of diagnosis and in a subset of patients (*n* = 96) at 6 ± 3-month follow-up.

The control collective consisted of healthy postpartum women with confirmed normal cardiac function (echocardiography, LVEF > 55 %, *n* = 19) in the first postpartum week when also plasma and serum probes were collected.

### Medication

Standard medication for heart failure was applied upon diagnosis and reported. A positive record with the healing attempt using bromocriptine therapy (BR therapy) was noted if a patient obtained bromocriptine according to the protocol published in our pilot study [20] which is based on an efficient suppression of Prolactin by 2.5–5 mg bromocriptine per day for at least 4 weeks together with beta-blocker and angiotensin-converting enzyme (ACE) inhibitor/angiotensin-receptor-blockers (ARBs) or other heart failure therapy according to the guidelines. Patients who did not obtain this bromocriptine therapy protocol but got the standard therapy for heart failure according to the guidelines were defined as the non-BR group. There was no randomization of patients to either group.

### Blood tests

Blood samples were collected at the time point of first diagnosis (baseline) and at the follow-up visit (6 ± 3 months after diagnosis) in S-Monovette^®^ tubes containing ethylenediaminetetraacetic acid (EDTA) or clot activator, respectively. Plasma and serum were separated by centrifugation at 1,500 rpm for 10 min. Aliquots were stored at −80 °C for future analysis. Laboratory workup was performed as routine investigation by hospital laboratories for Prolactin (Prolactin kit, Roche), N-terminal pro-brain natriuretic peptide (NT-proBNP), C-reactive protein (CRP), Thyroid stimulating hormone (TSH), total cholesterol, Troponin T (TnT), Creatine kinase (CK), Hemoglobin (Hb), full blood count, liver function, and Creatinine.

### Analysis of serum asymmetric dimethylarginine (ADMA)

Concentrations of serum ADMA were determined by gas chromatography-tandem mass spectrometry (GC-tandem MS) [8].

### Measurement of Cathepsin D

Serum Cathepsin D activity was evaluated with the Sensolyte 520 Cathepsin D Assay Kit (MoBiTec) as previously described [10].

### RNA extraction and miRNA expression analysis in the plasma by TaqMan MicroRNA Assay

Total RNA extraction was performed with the miRNeasy kit (Qiagen). Taqman methods were used to assess miRNA expression as previously described [9]. Briefly, RNA from 100 μl of serum was reverse transcribed to cDNA with the Taqman microRNA Reverse Transcription kit and the Taqman microRNA assay stem loop primers (Applied Biosystems). Resulting cDNAs were used for quantitative real-time PCR using Taqman microRNA assay and Taqman universal PCR master mix reagents (Applied Biosystems). Thermal cycling was performed on an Applied Biosystem 7900 HT detection system (Applied Biosystems). The relative miRNA levels were then normalized to two spikes-in miRNAs: cel-miR-39 and cel-miR-238 (Applied Biosystems).

### Analysis of outcome

After follow-up at 6 ± 3 months, patients were classified as described previously [5, 20, 23]. In brief, patients were classified as improvers (IMP) if LVEF increased by 10 absolute percent units or if NYHA improved by one class. Patients were classified as non-improvers (NIMPs) if they showed at the follow-up visit any of the parameters such as an LVEF < 35 %, failed to improve LVEF by 10 absolute units, remained at a NYHA functional class of III/IV or obtained heart transplantation or had died. Full recovery was defined as reaching an LVEF of ≥55 % and NYHA class I to II.

### Statistical analysis

Database management and statistical analyses were performed with SAS software, version 9.2 statistical program (SAS, Institute Inc., Cary, North Carolina, USA). Continuous data were expressed as mean ± SD or median and range. Comparison of means and proportions between sub-groups at baseline was performed by independent *t* test and Chi-square statistics, or Fisher exact test where necessary, respectively. Wilcoxon rank-sum test was used if data were not normally distributed. Significance was assumed at a two-sided value of *p* < 0.05.

## Results

### Onset and diagnosis of PPCM

Between 2004 and 2012 176 patients with suspected PPCM were reported to the German PPCM registry of whom a total of 115 patients matched the diagnostic criteria defined by Sliwa et al. [24] while 61 patients did not meet the diagnostic criteria, i.e., EF ≤ 45 % and absence of previously known cardiomyopathy. Mean LVEF at the time of diagnosis was 27 ± 9 %. LV dilatation (LVEDD > 56 mm) was present in 72 % (54/75) indicating that LV was not dilated in 28 % of patients at the time of diagnosis. All other clinical and serum parameters of all patients if available are indicated in supplementary material Table S1.

**Fig. 1:**
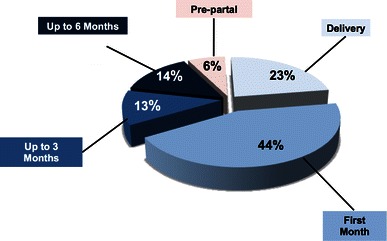
Time of diagnosis in relation to delivery in PPCM patients *n* = 90

Records on the time of diagnosis in relation to delivery, i.e., the last month of pregnancy, at delivery, the first month, 2–3 months, or later than 3 months after delivery were available from 90 patients. Most patients were diagnosed at delivery or in the first postpartal month (Fig. 1). Diagnosis prior delivery was made in 6 % (5/90 patients) between gestational week 28 and 36 followed by delivery within 1 day to 4 weeks. One patient with the anti-phospholipid syndrome developed PPCM after a miscarriage in the second trimester during the first pregnancy. A second patient with lupus erythematosus developed PPCM after a miscarriage in the first trimester. The median gravity was 2 (range 1–11) and the median parity was 2 (range 0–9) (supplementary material Table S1), with 36 % (35/96) being primipara.

### Risk factors and co-morbidities in PPCM patients

Information on risk factors and co-morbidities from PPCM patients were compared to healthy postpartum controls (*n* = 19, mean LVEF of 62 ± 4 %, range: 57–70 %) and, if available, to data from the general population in Germany (Table 1). PPCM patients tended to be older than the control collective and the average child-bearing woman in Germany (Table 1). Parity in PPCM patients was higher than in women of the control collective but similar to the overall parity in women in Germany (Table 1). The incidence of cesarean section (C-section) was significantly higher in PPCM patients compared to controls (*p* < 0.01), albeit also our control collective had a higher rate compared to the overall rate in Germany (Table 1). Emergency C-section was performed in 12.5 % (8 of 64 C-sections) of PPCM patients. We excluded patients with pregnancy-induced hypertensive disorders (HT: hypertension, preeclampsia and/or HELLP: HELLP-Syndrome: Hemolysis Elevated Liver enzymes Low Platelet count) in our postpartum controls, so valid comparison for this parameter is only possible to the overall frequency in Germany, which shows that the frequency of HT in our PPCM collective is higher (Table 1). There was a tendency for more smokers and more pathologic conditions of thyroid gland, while the incidence in gestational diabetes was similar in PPCM patients compared to controls and the overall population in Germany (Table 1). Previous chemotherapy due to childhood malignancies was reported in 1 of the 115 patients and in none of the controls.

**Table 1:** Comparison of factors for lifestyle, cardiovascular risk and pregnancy related conditions between PPCM patients, healthy postpartum controls and the general population in Germany

	PPCM	Controls	German statistical data
Age (years) (mean ± SD)	34 ± 6 (*n* = 113)	29 ± 5 (*n* = 19)	30^a^
Parity Median (range)	2 (0–9) (*n* = 97)	1 (1–3) (*n* = 19)	1.9^a^
Twin pregnancy	15 % (*n* = 17/110)	11 % (*n* = 2/19)	1.6 %^b^
C-section*	68 % (*n* = 64/94)	26 % (*n* = 5/9)	31.9 %^a^
Pregnancy-induced hypertensive disorders	45 % (*n* = 50/112)	0 % (*n* = 0/19)	1–5 %^c^
Smoking	45 % (*n* = 32/71)	26 % (*n* = 5/19)	25 %^a^
Pathologic condition of thyroid gland	23 % (*n* = 16/69)	11 % (*n* = 2/19)	
Gestational diabetes	7 % (*n* = 8/115)	11 % (*n* = 2/19)	3.7 %^d^
Tocolysis	4 % (*n* = 5/115)	0 % (*n* = 0/19)	

*C-section* cesarean section

* *p* < 0.01 PPCM vs. healthy postpartum controls

Data for the overall population in Germany were obtained from ^a ^the Federal Statistical Office (https://www.destatis.de/DE/Publikationen/Thematisch/Bevoelkerung), ^b^ http://de.statista.com/statistik/daten/studie/1281/umfrage/anzahl-der-zwillingsgeburten-in-deutschland-2006/, ^c^ http://www.hochdruckliga.de, ^d^ http://www.deutsche-diabetes-gesellschaft.de/

### Biomarker profiles in patients with acute PPCM compared to healthy postpartum controls

**Fig. 2:**
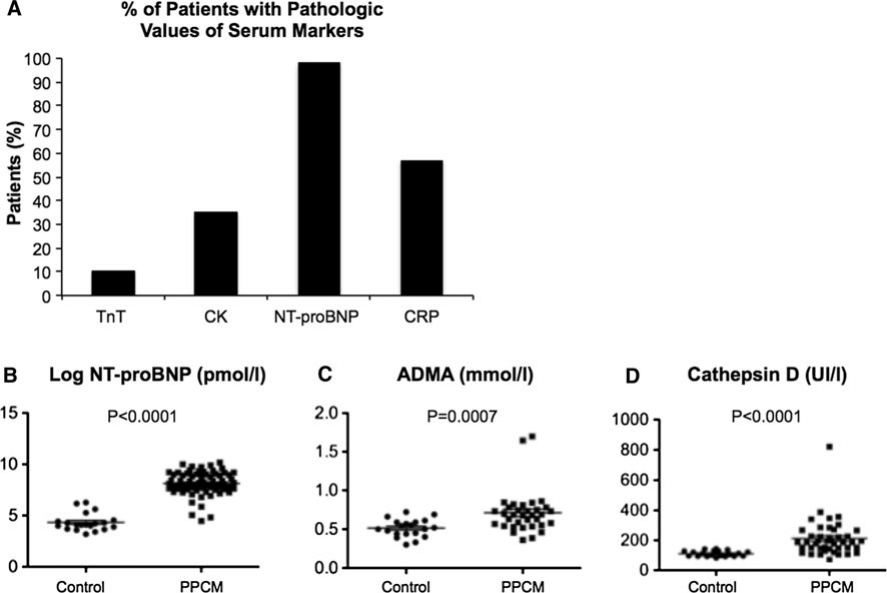
**a** Percentage of PPCM patients with normal (TnT normal <0.01 μg/l, NT-proBNP normal in women <146 μg/l, CK normal in women <145 U/l, CRP normal <8 mg/l) and with pathophysiologic relevant serum levels of TnT (*n* = 49), CK (*n* = 63), NT-proBNP (*n* = 69) and CRP (*n* = 72) at the time of diagnosis. Baseline serum levels of **b** NT-proBNP (PPCM, *n* = 69; control, *n* = 19), **c** ADMA (PPCM, *n* = 34; control, *n* = 19) and **d** Cathepsin D (PPCM, *n* = 43; Control, *n* = 19). *p* value compares PPCM vs. healthy postpartum controls

Serum analyses showed that TnT was within normal range in the majority of PPCM patients (Fig. 2a, supplementary material Table S1). CK was up-regulated in 37 % of PPCM patients but is also frequently up-regulated in healthy peripartum women [17] (Fig. 2a, b, supplementary material Table S1). Likewise, CRP was increased in the majority of healthy postpartum controls and in several PPCM patients but was normal in others (Fig. 2a, supplementary material Table S1). NT-proBNP, a marker for heart failure, was increased in almost all PPCM patients but rarely in healthy postpartum controls (Fig. 2a, b, supplementary material Table S2). ADMA is a known marker for hypertensive complications in pregnancy such as preeclampsia [18], a pregnancy complication frequently present in our PPCM collective. Cathepsin D has been shown to be elevated in a small collective of PPCM patients [10]. Serum levels of ADMA and Cathepsin D activity were significantly higher in PPCM patients compared to postpartum controls, but both markers showed a broad range of overlapping values between the two groups (Fig. 2c, d, supplementary material Table S2). Stratification of ADMA serum levels in Quartiles (cut-off values: 1st Quartile: 0.52 μmol/l; 2nd Quartile: 0.59 μmol/l; 3rd Quartile: 0.69 μmol/l; 4th Quartile: 0.85 μmol/l) showed that the portion of the patients with a full recovery gradually decreased from the 2nd to the 4th quartile, albeit with no significant differences (supplementary material Fig. 1). We recently reported that 16 kDa Prolactin induces miR-146a expression in endothelial cells and showed that it is up-regulated in a small collective of PPCM patients [9].

**Fig. 3:**
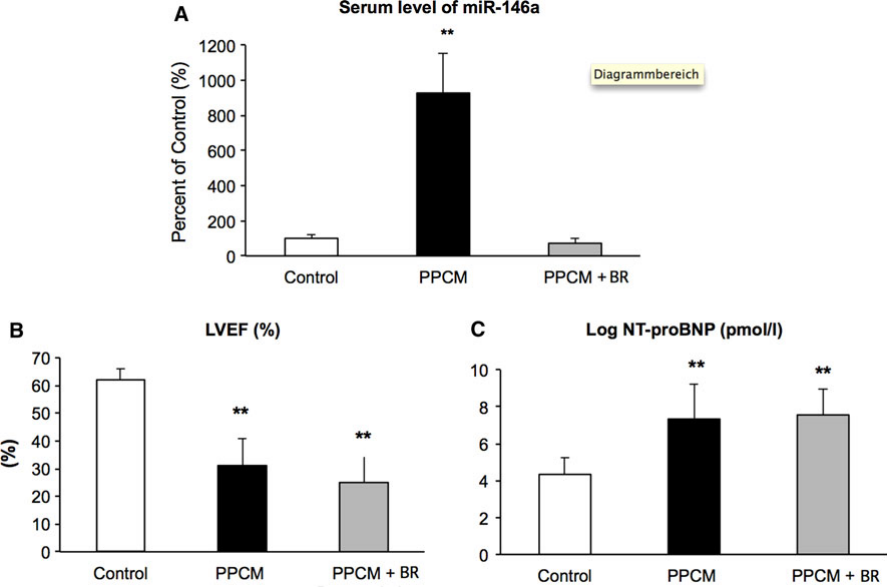
**a** *Bar graph* displaying elevated serum levels of miR-146a of patients with acute PPCM without bromocriptine (*n* = 40) compared with healthy postpartal controls (*n* = 19) and with acute PPCM patients who obtained already bromocriptine (BR) for 3–7 days when blood samples were taken (*n* = 17). **b** *Bar graph* showing baseline LVEF and **c** NT-proBNP in the same PPCM patients with or without early bromocriptine (BR) treatment. **p = 0.001 vs. Control

Here we observed increased serum levels of miR-146a in 57 PPCM patients at baseline compared with 19 pregnancy-matched healthy postpartal women (Fig. 3a). Furthermore, we found that PPCM patients who were already treated for 3–7 days with bromocriptine before the initial diagnosis of PPCM was made and the baseline blood sample was collected displayed significantly lower miR-146a levels as compared to not-treated patients (Fig. 3a), while LVEF and NT-proBNP levels were not yet changed (Fig. 3b, c).

### Follow-up analyses in PPCM patients

Follow-up data for 6 ± 3 months on LVEF were available for 96 patients while 19 patients were lost to follow-up. The mean follow-up LVEF increased from 27 ± 9 to 47 ± 19 %. The majority of patients were defined as improvers (IMP, 85 %, 82/96), which according to our pre-specified criteria (see material and method section) include partial recovery and full recovery. In 47 % (45/96) of patients, full recovery with an LVEF ≥ 55 % and NYHA class I to II was observed. Of the 15 % (14/96) patients classified as non-improvers (NIMP), 4 patients displayed a follow-up LVEF < 30 %, 1 patient needed a left ventricular assist device (LVAD), and 7 patients obtained heart transplantation (HTX). Two patients died, 1 of them after transplantation and 1 due to sudden cardiac death. Eight patients obtained an implantable cardioverter-defibrillator (ICD), 5 patients in the IMP and 3 patients in the NIMP group. Defibrillator life vests were used in 2 patients in the IMP (both patients recovered and did not need ICDs) and 1 patient in NIMP group (ICD was implanted after an episode of ventricular fibrillation).

Subsequently, we compared baseline characteristics between IMP and NIMP and found that in NIMP baseline LVEDD was larger and baseline LVEF was lower compared to IMP (*p* < 0.0001, Table 2). In fact baseline LVEF in all NIMPs was ≤25 %. In addition, baseline AST and GGT were higher in NIMP compared to IMP (*p* = 0.006 and *p* = 0.004, Table 2). In addition, no significant differences between IMP vs. NIMP were observed with regard to co-morbidities such as smoking, gestational diabetes, mode of delivery, older age, low Hb and hypothyroidism (Table 2). Significantly more patients in the IMP group reported pregnancy-associated hypertension compared to NIMP (HT in IMP: 49 % 40/82 vs. HT in NIMP: 7 %, 1/14, *p* = 0.009, Table 2).

**Table 2:** Baseline characteristics improvers (IMP) vs. non-improvers (NIMP)

Variables	Number of patients	IMP	NIMP	*p* value IMP vs. NIMP
Age (years) (mean ± SD)	95	34 ± 6 (*n* = 81)	33 ± 4 (*n* = 14)	0.42
Gravida Median (range)	83	2 (1–11) (*n* = 72)	2 (1–3) (*n* = 11)	0.74
Parity Median (range)	83	2 (0–8) (*n* = 70)	2 (1–3)(*n* = 13)	0.60
Hypertensive disorder in pregnancy	96	49 % (*n* = 40 of 82)	7 % (*n* = 1 of 14)	**0.009**
NYHA: *n* (%)	84			0.06
*n*: II		*n* = 13 (18 %)	*n* = 0 (0 %)	
*n*: III		*n* = 28 (40 %)	*n* = 2 (15 %)	
*n*: IV		*n* = 30 (42 %)	*n* = 11 (85 %)	
Heart rate bpm (mean ± SD)	60	94 ± 27 (*n* = 52)	104 ± 19 (*n* = 8)	0.16
LVEDD (mm) (mean ± SD)	66	59 ± 7 (*n* = 55)	70 ± 8 (*n* = 11)	**0.002**
LVEF (%) (mean ± SD)	96	28 ± 9 (*n* = 82)	17 ± 5 (*n* = 14)	**<0.0001**
Prolactin (ng/ml), median (range)	45	13.2 (0.7–258) (*n* = 40)	128 (4.7–202) (*n* = 5)	N/A
CRP (mg/l), median (range)	67	9 (0.4–219) (*n* = 57)	17 (0.1–147) (*n* = 10)	0.52
Cathepsin D	38	191 (72–821) (*n* = 36)	171 (143–199) (*n* = 2)	N/A
ADMA (μmol/l)	30	0.68 (0.36–1.70) (*n* = 28)	0.69 (0.54–0.84) (*n* = 2)	N/A
Log NT-proBNP (pmol/ml) (mean ± SD)	60	8.1 ± 1.1 (*n* = 54)	8.2 ± 0.8 (*n* = 6)	N/A
Total cholesterol mg/dl), median (range)	36	218 (7.8–345) (*n* = 28)	186 (92–451) (*n* = 8)	0.14
AST (U/l), median (range)	64	28 (12–263) (*n* = 54)	445 (26–197) (*n* = 10)	**0.006**
ALT (U/l), median (range)	63	26.5 (8–441) (*n* = 54)	95 (23–180.5) (*n* = 9)	0.09
GGT (U/l), median (range)	48	24.5 (4–81) (*n* = 40)	60.6 (32–157) (*n* = 8)	**0.004**
Creat (μmol/l), median (range)	62	74 (1–123) (*n* = 54)	90.5 (0.8–114) (*n* = 8)	0.45
TSH (mU/l), median (range)	63	1.8 (0.01–10.9) (*n* = 53)	2.1 (0.89–6.4) (*n* = 10)	0.46
CK (U/l), median (range)	57	63 (14–1654) (*n* = 50)	38 (0.6–62) (*n* = 7)	0.24
TnT (μg/l), median (range)	42	0.02 (0.01–70) (*n* = 36)	0.01 (0.01–0.02) (*n* = 6)	N/A
Hb (g/dl) (mean ± SD)	70	11.7 ± 1.7 (*n* = 62)	10.6 ± 2.5 (*n* = 8)	1.00

Data for all patients with follow-up classified as IMP or NIMP are provided at baseline with the number of patients (*n*) analyzed behind each values. *p* value compare IMP vs. NIMP, parameters with N/A indicated that meaningful comparison was not possible due to low number of data sets

### Clinical presentation and serum marker profiles in PPCM patients with and without hypertensive conditions during pregnancy

The differences in the incidence of hypertensive conditions between IMP and NIMP prompted us to evaluate whether patients with HT may display a different clinical presentation at baseline compared to patients without HT. As shown in the supplementary material Table S3 only NT-proBNP levels were slightly but significantly higher in PPCM patients with HT compared to PPCM without HT conditions during pregnancy (*p* = 0.039). All other parameters investigated at the time point of PPCM diagnosis such as gravida, para, NYHA, blood pressure (high blood pressure in the hypertensive group was diagnosed during pregnancy and treated), LV dimension, LV function, heart rate, ADMA, Prolactin and total cholesterol and other serum markers did not differ between the two groups (supplementary material Table S3).

### Recovery rate in PPCM patients with a positive family history of cardiomyopathy

A positive family history of cardiomyopathy (PPCM, DCM, sudden death, arrhythmias in first degree relatives) was reported in 16.5 % (19/115) of PPCM patients with a baseline LVEF of 25 ± 6 %. Recovery rate of patients with positive family history of cardiomyopathy was similar to patients without such a history of follow-up data: 82 % (14/17) improved with complete recovery in 47 % (8/17). There is a tendency that more of these patients (18 %, 3/17) needed a HTX compared to patients with no reported family history of cardiomyopathies (5 %, 4/79), albeit no significancy is reached.

### Follow-up related to medical therapy

Records on medical history implemented after diagnosis were available in all 96 patients with follow-up data. With regard to standard therapy for heart failure, our analysis revealed that more IMP obtained beta-blockers and/or ACE inhibitors/angiotensin-receptor-blockers (ARB) compared to the NIMP (Table 3). In addition, a high percentage of patients (67 %, 64/96) obtained bromocriptine according to the protocol we have published in our pilot study [20] (BR group, methods section), the overall percentage of IMP and of NIMP who obtained bromocriptine is shown in Table 3. There was a significantly higher percentage of patients classified as IMP (92 %, 59/64) and a lower percentage classified as NIMP (8 %, 5/64) in the BR group compared to the non-BR group (IMP: 72 %, 23/32, NIMP: 28 %, 9/32). The percentage of patients experiencing full recovery (LVEF ≥ 55 % with NYHA class I to II) was similar in both groups with no difference in baseline characteristics (supplementary material Table S4). Further analyses revealed that the majority of patients, 96 % (55/57) who had obtained all three drugs, beta-blockers, ACE inhibitors/ARBs and bromocriptine were IMPs. In turn, only 14 % (2/14) of NIMP patients had obtained the triple therapy.

**Table 3:** Medication in PPCM of patients with regard to recovery

	IMP % (*n* = 82)	NIMP % (*n* = 14)	Full recovery % (*n* = 45)	*p* value IMP vs. NIMP
Bromocriptine	72 (*n* = 59)	35 (*n* = 5)	67 (*n* = 30)	0.013
Beta-blockers	95 (*n* = 78)	50 (*n* = 7)	93 (*n* = 42)	0.0001
Bisoprolol	29 (*n* = 24)	29 (*n* = 4)	24 (*n* = 11)	
Metoprolol	51 (*n* = 42)	21 (*n* = 3)	55 (*n* = 24)	
Carvedilol	13 (*n* = 11)	0	11 (*n* = 5)	
Esmolol	1 (*n* = 1)	0	2 (*n* = 1)	
ACE Inhib or ARB	93 (*n* = 76)	71 (*n* = 10)	91 (*n* = 41)	0.04
ACE Inhib	84 (*n* = 69)	64 (*n* = 9)	80 (*n* = 36)	0.16
ARB	11 (*n* = 9)	8 (*n* = 1)	14 (*n* = 6)	0.97
Ramipril	73 (*n* = 58)	54 (*n* = 7)	68 (*n* = 30)	
Enalapril	8 (*n* = 7)	0	11 (*n* = 5)	
Lisinopril	1 (*n* = 1)	0	0	
Candesartan	6 (*n* = 5)	7 (*n* = 1)	7 (*n* = 3)	
Irbesartan	1 (*n* = 1)	0	0	
Valsartan	2 (*n* = 2)	0	4 (*n* = 2)	
Telmisartan	1 (*n* = 1)	0	2 (*n* = 1)	
MRA	65 (*n* = 53)	57 (*n* = 8)	56 (*n* = 25)	0.81
Eplerenone	7 (*n* = 6)	7 (*n* = 1)	7 (*n* = 3)	
Spironolactone	57 (*n* = 47)	50 (*n* = 7)	45 (*n* = 22)	
Diuretics	76 (*n* = 62)	86 (*n* = 12)	65 (*n* = 29)	0.51
Loop-Diuretics	70 (*n* = 57)	71 (*n* = 10)	53 (*n* = 24)	
Thiazide	13 (*n* = 10)	15 (*n* = 2)	18 (*n* = 8)	
Digitalis	5 (*n* = 4)	21 (*n* = 3)	4 (*n* = 2)	0.06
Digoxin	2 (*n* = 2)	21 (*n* = 3)	2 (*n* = 1)	
Digitoxin	2 (*n* = 2)	0	2 (*n* = 1)	

Medical history for all patients with follow-up, classified as IMP or NIMP, are provided with the number of patients (*n*) analyzed behind each values. *p* value compare IMP (full and partial recovery) vs. NIMP, parameters

## Discussion

This report documents the first prospective multicenter registry of 115 newly diagnosed PPCM patients in Germany spanning a recent observation period from 2004 to 2012. It is one of the largest prospective cohort studies of PPCM patients on contemporary management and shows the association of baseline LVEF and pregnancy-associated hypertension for prognosis. The study supports the idea that PPCM might be provoked by pathogenic factors including pregnancy-induced hypertension and smoking and it elucidates the potential value of markers such as NT-proBNP, Cathepsin D, ADMA and miRNA-146a as markers for diagnosis and disease monitoring. Moreover, increased plasma levels of Cathepsin D, ADMA and miRNA-146a support the hypothesis that a circuit involving Prolactin cleavage and subsequent endothelial dysfunction acts as a major pathophysiological concept for this disease. Finally, it is the largest cohort treated with the novel disease specific therapy concept using the Prolactin blocker bromocriptine in addition to the standard therapy for heart failure aiming to block potential adverse effects of the angiostatic 16 kDa Prolactin. It supports a potential benefit of a treatment concept with bromocriptine, beta-blockers and ACE inhibitors/ARBs but points also out that it may not be sufficiently effective in all patients, especially in PPCM patients with very low baseline EF.

Most patients were diagnosed at delivery or in the first postpartum month, an observation that is similar to findings from studies on a South African population [22] and from a study from Haiti [4] documenting almost no patient with signs of heart failure during the pre-partum period but different to reports from PPCM collectives in the USA [3] and Japan [12]. However, it is likely that in some patients symptoms of heart failure were present earlier, i.e., during pregnancy prior the initial diagnosis. This feature could not be addressed properly in this study but warrants random screening efforts in pregnant women, especially in such with a high risk profile including smoking, pregnancy-associated hypertension, twin pregnancy and older age. In addition, the observation that onset of PPCM occurred also in patients after fetal loss earlier in pregnancy suggests that PPCM may not be linked exclusively to full-term pregnancies but to changes in the maternal physiology around the time of delivery.

Numerous factors predicting a higher risk for developing PPCM have previously been proposed such as older age, multiparty, obesity, smoking, delayed diagnosis, being of African descent and preeclampsia [3, 19, 21]. In our study, a history of smoking was present in almost half of PPCM patients while only one forth of our control postpartum collective and of women in the same age group in accordance to data of the “Statistische Bundesamt” (http://www.gbe-bund.de/) were smokers suggesting that smoking may increase the risk for PPCM. The mean age of PPCM patients was higher compared to the average age of child-bearing mothers in Germany and to the average age of our control collective which is in line with previous reports that older age appears to be a risk factor for the disease. Two third of PPCM patients developed the disease in a subsequent pregnancy, and the percentage of twin pregnancies, albeit similar in our control collective, was also higher in the PPCM collective compared to the overall frequency in Germany, confirming previous observation that the risk for PPCM increases with multiple parities and twin pregnancies [21, 24]. Interestingly, gestational diabetes was not more frequent in PPCM compared to the frequency in normal pregnancies in Germany. The number of patients with tocolysis or childhood chemotherapy was too low so no statement on these risk factors can be made in our collective. Taken together, this risk factor analysis further underline the importance of an adequate cardiac monitoring during pregnancy especially in women with pregnancy-associated hypertension, older age, or smokers.

The mode of delivery is also discussed as a potential risk factor and indeed we observe that the frequency of cesarean section was doubled in PPCM patients compared to the overall frequencies reported in Germany. We cannot distinguish whether C-section may be a risk factor or whether the clinical condition of PPCM patients may more frequently be an indication for a C-section. However, it can be speculated that a higher degree of cell traffic between the baby and the mother takes place in C-section deliveries, which may increase the incidence of PPCM based on immune reaction.

Recently, several studies reported that PPCM occurred in patients, who had a positive family history of cardiomyopathies suggesting that the pregnancy/peripartum stress may have demasked a genetic form of cardiomyopathy [14, 25]. Indeed, 16.5 % of PPCM patients in our registry reported a positive family history of cardiomyopathy supporting the idea that genetic factors may be involved in some PPCM patients. We observed that the number of HTX was more than 3 times higher in these patients, albeit not significant but nevertheless suggesting that they may be more refractory to medical therapy and tend to have a more severe course of disease [15, 16]. This observation if confirmed in larger collectives could be important for risk stratification and management of these patients. However, the majority of our patients had no family history of cardiomyopathies and displayed an apparently unremarkable cardiac condition prior to pregnancy suggesting that a genetic predisposition towards heart failure appears to be rather rare in this cohort.

One of the most striking findings from this analysis is the poor prognosis of patients with very low baseline LVEF (<25 %) compared to patients with higher baseline LVEF despite fairly high adherence to guideline-indicated pharmacological therapy, although the optimum doses were not reached in all cases. This observation strongly suggests that initial LVEF is an important determinant of prognosis, a notion that is in line with observations in PPCM patients from South Africa [5, 21, 23] and from the USA [7]. However, also in our cohort patients with baseline LVEF < 25 % had the potential to recover. Therefore, we agree with Goland et al. [6] and discourage from using baseline LVEF alone as an indication for premature use of aggressive therapy such as assist device implantation or HTX. In contrast with the African collective [5], we did not find a correlation between poor outcome and high NT-proBNP levels. It is also important to note that more IMPs obtained beta-blockers and ACE inhibitors/ARBs compared to the NIMPs, albeit this observation may also be due to better hemodynamic conditions of the IMP in general allowing to add beta-blocker therapy, whereas the non-improvers more often presented with low blood pressure and bradycardia impeding the application of beta-blockers.

One reason for poor outcome in PPCM could be skepticism of physicians to consider the diagnosis of PPCM in patients with symptoms of heart failure in the peripartal phase who had no previous history of heart disease. Specific biomarkers for PPCM are scarce. Typical markers for cardiac injury or inflammation are either frequently not up-regulated, i.e., TnT or are up-regulated also in healthy postpartum women, i.e., CK [17] and CRP. Although NT-proBNP as a classical, yet unspecific biomarker for heart failure was elevated in almost all patients, it is not suited to differentiate between PPCM and other causes of heart failure.

**Fig. 4:**
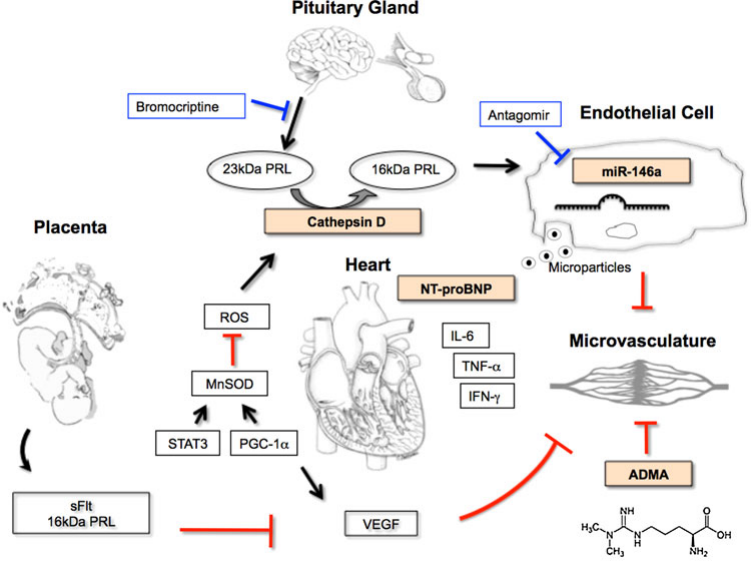
Scheme depicting the pathophysiological circuits in PPCM. Note that Cathepsin D, NT-proBNP, miR-146a and ADMA were tested as potential biomarkers of PPCM in this study. The scheme illustrates the release of Prolactin from the pituitary gland towards the end of pregnancy, which under conditions of enhanced oxidative stress (ROS) in the myocardium is proteolytically cleaved to a 16 kDa fragment by Cathepsin D. In healthy myocardium this process is prevented by antioxidative factors such as MnSOD, which is regulated by certain transcription factors such as STAT3 and PGC1-α. The 16 kDa Prolactin leads to increased miR-146a expression in endothelial cells, which exerts angiotoxic effects and impairs via an exosome-mediated paracrine fashion the metabolic activity of cardiomyocytes and the crosstalk between endothelial cells and cardiomyocytes via down-regulation of ErbB4. An imbalance between VEGF and the soluble VEGF receptor sFlt as well as increased ADMA levels add to the anti-angiogenetic effects of the 16 kDa Prolactin-miR-146a axis. Blocking Prolactin with bromocriptine or inhibition of the 16 kDa Prolactin effector miR-146a with antagomirs prevents or attenuates detrimental effects of 16 kDa Prolactin. Enhanced levels of pro-inflammatory cytokines such as IL-6, TNF-α and IFN-γ as previously reported point to an additional inflammatory component within the pathomechanisms of PPCM. NT-proBNP as an unspecific marker of heart failure is increased in almost all PPCM patients. *ROS* reactive oxygen species, *MnSOD* manganese Superoxide Dismutase, *STAT3* signal transducer and activator of transcription 3, *PGC-1α* peroxisome proliferator-activated receptor gamma, coactivator 1 alpha, *sFlt* soluble fms-like tyrosine kinase-1, *VEGF* vascular endothelial growth factor, *IL-6* interleukin-6, *TNF-α* tumor necrosis factor-α, *IFN-γ* interferon-γ, *ADMA* asymmetric dimethylarginine

Therefore, we specifically investigated potential biomarkers that would be associated with suspected pathophysiological mechanisms present in PPCM. This profile includes beside the classical biomarker for heart failure, NT-proBNP, novel markers, which were driven from findings of experimental studies such as Cathepsin D, ADMA and miR-146a. For Cathepsin D, we showed that it is activated in the myocardium in an animal model of PPCM in response to conditions of enhanced oxidative stress during pregnancy in case of defective anti-oxidative mechanisms. Activated Cathepsin D in turn leads to proteolytic cleavage of nursing hormone Prolactin into a 16 kDa fragment that exerts anti-angiogenic effects [10]. The observation that Cathepsin D activity was significantly higher in PPCM patients compared to postpartum controls not only suggests Cathepsin D as a potential biomarker for PPCM but also supports the findings from the experimental studies (Fig. 4). More recently, we reported that 16 kDa Prolactin induces the expression of miR-146a in endothelial cells and showed that miR-146a is mediating most of the anti-angiogenic effects of 16 kDa in endothelial cells [9]. In addition, 16 kDa Prolactin triggers the release of miR-146a-loaded exosomes from endothelial cells which are absorbed by cardiomyocytes where they substantially alter gene expression, i.e., ErbB4 and as a consequence metabolic activity [9]. We reported a specific increase of miR-146a in the serum of a small group of PPCM patients compared to healthy postpartum controls and patients with dilated cardiomyopathy [9], a feature that fits well to our other observation that endothelial microparticles are highly specifically increased in serum of PPCM patients [26]. Here we confirm the specific up-regulation of miR-146a in our collective of PPCM patients compared to healthy postpartum controls. In addition, patients with early bromocriptine treatment displayed normalized miR-146a serum levels while cardiac function and the heart failure marker NT-proBNP were not different to untreated patients at baseline. This observation further supports the pathophysiologic connection of miR-146a with 16 kDa Prolactin in the human PPCM disease and suggests that bromocriptine treatment is highly efficient to clear the system from 16 kDa Prolactin and its downstream effectors.

ADMA, a marker of endothelial dysfunction and a consequence of oxidative stress, was significantly higher in serum from PPCM patients compared to healthy postpartum women thereby supporting the idea of substantial endothelial dysfunction in PPCM. Moreover, this observation adds ADMA to the pool of potential biomarkers for PPCM. Elevated ADMA levels have been reported to be associated with an enhanced risk of developing preeclampsia [18], a feature that is quite interesting in the view that hypertensive complications such as preeclampsia were quite prominent in our PPCM collective. However, ADMA has also been shown to be elevated in patients with ischemic and dilated cardiomyopathy limiting its potential to differentiate between different causes of heart failure [1].

In summary, the biomarker profile described in this study, i.e., Cathepsin D, miR-146a, ADMA and NT-proBNP seem to support the idea that a circuit of unbalanced oxidative stress, activated Cathepsin D and subsequent cleavage of Prolactin in its 16 kDa form is a driving force for the development of PPCM. This concept is further supported by the finding that baseline Prolactin levels were higher in patients with a poor outcome in our cohort, but the numbers are too low to be conclusive. However, the highest recovery rate was observed among patients who obtained the Prolactin blocker bromocriptine in addition to beta-blockers and ACE inhibitors/ARBs.

Recent experimental and clinical studies [15] reported that pregnancy-induced hypertensive conditions such as preeclampsia may be a risk factor for PPCM by providing an extremely anti-angiogenic environment which may include sFlt1 and 16 kDa Prolactin. Indeed, high levels of sFlt-1 after delivery may be an additional factor that triggers PPCM after preeclampsia [15], a feature that is currently investigated in larger PPCM collectives. Similar to a PPCM collective described in Japan [12], we found a high incidence of hypertensive conditions. In fact, one patient with severe preeclampsia, who had documented normal cardiac function prior delivery, developed PPCM after delivery further emphasizing that preeclampsia may sensitize for PPCM. Also similar to the Japanese collective [12] is our observation that PPCM with concomitant hypertension was associated with a higher recovery rate. This feature is in accordance with the association of elevated blood pressure and a better outcome in patients with acute heart failure [13], and will be investigated more closely in the future. In the African cohort, study patients with >160 mmHg systolic and >110 mmHg diastolic blood pressure were always excluded as the authors felt that hypertensive heart disease in particular in African women can per se lead to systolic dysfunction via a possibly different pathomechanism [22]. The German cohort is therefore not fully comparable with the African cohort studies as the inclusion and exclusion criteria are different.

In contrast to most other studies on outcome in PPCM patients, a high percentage of patients in our cohort was treated with the Prolactin blocker bromocriptine in addition to standard treatment for heart failure [10, 11, 20]. Most of these patients obtained beta-blockers and ACE inhibitors/ARBs in addition to bromocriptine while most of NIMP patients did not obtain this drug combination. This observation suggests that patients obtaining the combination of these three medications may have a higher chance for recovery (partial and full recovery). In parallel to the general recommendations in all patients with systolic heart failure, beta-blockers and/or ACE inhibitors/ARBs seem to be highly beneficial and should always be considered early in the treatment of PPCM with uptitration to the maximum tolerated dose. The question whether bromocriptine on top of these standard therapeutics has an additional benefit cannot be answered by this observational trial. Therefore, it is necessary to test the BR therapy concept in controlled randomized multicenter trials as the one that is currently performed in Germany (randomization of 60 PPCM patients to BR therapy or no BR therapy, study registered at ClinicalTrials.gov, study number: NCT00998556). Moreover, since not all patients profited equally from the above-mentioned triple drug therapy concept, further characterization, such as genetics and risk factor profiles of different PPCM etiologies, are required for better management and risk stratification in these patients.

### Limitations of our study

Limitations to this study are that 19 PPCM patients with baseline data were lost to follow-up and that the data analyzed including the echocardiographic data were obtained from records provided by physician without quality control. Minimal requirement for enrolment in this registry was baseline LVEF and close relationship to pregnancy (pregnant or delivery up to 6 months ago), not all patients agreed to provide blood probes and additional clinical data, a reason why not all other data sets are complete in this registry. For CRP and TnT, only standard and no novel high sensitive assays were used. It is important to note that the results regarding medications, specifically bromocriptine treatment, derive from a prospective observational analysis and are not based on a randomized and/or placebo-controlled clinical trial.

In conclusion, the observations gained from this first Western European prospective cohort study show similarities with the clinical profile described in PPCM patients in the US with low mortality and a relative high incidence of recovery, a strong association with gestational hypertension as a risk factor for the disease and low baseline EF associated with poor outcome. In addition, it supports the idea that familial predisposition may be a risk factor in some patients. It discovered a biomarker profile that supports the hypothesis of a circuit including unbalanced oxidative stress, Prolactin cleavage into the anti-angiogenic 16 kDa form and subsequent severe endothelial damage and dysfunction as a major driving force for PPCM. This highly specific biomarker profile may help to distinguish PPCM patients at an early stage from healthy postpartum women. Finally, the high recovery rate in the present PPCM collective is associated with the largest series using a novel therapy concept of a combination of beta-blockers, ACE inhibitors/ARBs and bromocriptine, a feature that encourages further testing of potential benefits of this treatment concept in randomized studies as outlined above.

## Electronic supplementary material

Below is the link to the electronic supplementary material.

Supplementary material 1 (DOC 144 kb)
