# Supplementary material for: Phenotyping and outcome on contemporary management in a German cohort of patients with peripartum cardiomyopathy
Source: Basic Res Cardiol. 2013 Jun 28;108(4):366. doi: 10.1007/s00395-013-0366-9 (PMC3709080; doi:10.1007/s00395-013-0366-9)
Supplement: Supplementary file 1 — Supplementary material 1 (DOC 144 kb) [file 395_2013_366_MOESM1_ESM.doc]

**Phenotyping and outcome on contemporary management in a German cohort of patients with peripartum cardiomyopathy**

^1^A. Haghikia#, ^1^E. Podewski#, ^2,3^E. Libhaber, ^1^S. Labidi, ^1^D. Fischer, ^1^P. Roentgen, ^4^D. Tsikas, ^4^J. Jordan, ^5^R. Lichtinghagen, ^6^C.S. von Kaisenberg, ^7^I. Struman, ^7^N.Bovy ^2^K. Sliwa*, ^1^J. Bauersachs*, ^1^Denise Hilfiker-Kleiner*

^1^Department of Cardiology and Angiology, Medical School Hannover, Germany

^2^Hatter Cardiovascular Research Institute, Department of Medicine, Faculty of Health Sciences, University of Cape Town, South Africa

^3^School of Clinical Medicine, University of the Witwatersrand, Johannesburg, South Africa

^4^Department of Clinical Pharmacology, Medical School Hannover, Germany

^5^Department of Clinical Chemistry, Medical School Hannover, Germany

^6^Department of Gynecology and Prenatal Medicine, Medical School Hannover, Germany

^7^Unit of Molecular Biology and Genetic Engineering, GIGA, University of Liège, Liège, Belgium

# The first and second author contributed equally

* The three last authors contributed equally

Corresponding author:

Denise Hilfiker-Kleiner, PhD

Department of Cardiology and Angiology

Medical School Hannover

Carl-Neuberg-Str. 1

30625 Hannover, Germany

Phone: +49-511-532-2531, Fax: +49-511-532-3263

E-mail: Hilfiker.denise@mh-hannover.de

**Supplementary Data**

**Table 1:** Baseline characteristics in all PPCM patients

| Variables: | Number of patients | All patients, |
| --- | --- | --- |
| Age(years) (mean±SD) | 113 | 34± 6 |
| Gravida Median (range) | 95 | 2 (1-11) |
| Parity Median (range) | 97 | 2 (0-9) |
| NYHA: n, (%)  n: II  n: III  n: IV | 93 | n=14 (15%)  n=30 (32%)  n=49 (53%) |
| Heart rate bpm (mean±SD) | 67 | 96 ±27 |
| LVEDD (mm) (mean±SD) | 75 | 61 ± 8 |
| LVEF (%) (mean±SD) | 115 | 27 ± 9 |
| Prolactin (ng/ml)  Median (range) | 50 | 17 (0.7-258) |
| CRP (mg/l) Median (range) | 72 | 9.8 (0.1-219) |
| Total cholesterol mg/dl) Median (range) | 37 | 209 (7.8-451) |
| AST (U/l) Median (range) | 71 | 30 (12-263) |
| ALT (U/l) Median (range) | 70 | 28 (8-441) |
| GGT (U/l) Median (range) | 55 | 27 (4-603) |
| Creat (µmol/l) Median (range) | 66 | 75.5 (0.7-344) |
| TSH mU/l Median (range) | 69 | 1.9 (0.01-10.9) |
| CK (U/l) Median (range) | 63 | 62 (0.9-1654) |
| TnT (µg/l) Median (range) | 49 | 0.02 (0.01-70) |
| Hb(g/dl) (mean±SD) | 76 | 11.6 ±1.8 |

Data for all patients are provided at baseline with the number of patients (n) analyzed behind each values.

**Supplementary Table 2:** Serum levels of NT-proBNP. Cathepsin D and ADMA in PPCM at baseline in comparison with healthy postpartum controls.

|  | PPCM | Control | p-value* |
| --- | --- | --- | --- |
| NT-proBNP (median and range) | 3315 (875-26082), n=69 | 61 (24-531), n=19 | <0.0001 |
| Cathepsin D (median and range | 186 (72-821), n=43 | 109(82-144), n=19 | <0.0001 |
| ADMA (median and range | 0.68 (0.36-1.70), n=34 | 0.51 (0.30-0.72), n=19 | <0.0008 |

**Supplementary Table 3:** Baseline characteristics in PPCM of patients with and without hypertensive conditions during pregnancy

|  | PPCM with HT | PPCM without HT | p-value |
| --- | --- | --- | --- |
| Age (years)* | 33.1±6.4 (n=49) | 34.5± 5.7 (n=62) | 0.21 |
| Gravida Median(range) | 2(1-11) (n=43) | 2(1-9) (n=51) | 0.42 |
| Parity Median(range) | 2 (0-9) (n=43) | 2(1-8)(n=53) | 0.87 |
| NYHA functional class (I-IV)  (n) | I 1  II 4  III 17  IV 18 | I 0  II 12  III 16  IV 25 | 0.21 |
| Systolic blood pressure* (mmHg) | 119± 26 (n=24) | 116± 18 (n=32) | 0.84 |
| Diastolic blood pressure*  (mmHg) | 78± 19 (n=24) | 76± 15 (n=32) | 0.73 |
| Heart rate (bpm)* | 94± 29 (n=25) | 97± 24 (n=42) | 0.28 |
| Left ventricle, end-diastolic diameter (mm)* | 59.6± 8.1(n=33) | 62.2± 8.5(n=42) | 0.19 |
| Left ventricle, end-systolic diameter (mm)* | 49.9± 9.5(n=26) | 53.2± 8.7(n=31) | 0.13 |
| LVEF (%)* | 28.1± 8.8(n=50) | 25.5± 9.1(n=62) | 0.13 |
| ADMA (μmol/l) Median(range) | 0.73 (0.36-1.70)  (n=17) | 0.63 (0.39-0.86) (n=17) | 0.33 |
| Prolactin(ng/ml) Median(range) | 22(8-258)(n=19) | 10.3(1-202) (n=31) | 0.08 |
| Cathepsin D (% activity of control) Median(range) | 177(105-821) (n=23) | 192(72-388) (n=20) | 0.43 |
| CRP (mg/l) Median(range) | 18.5 (0.4-142) (n=31) | 9.6(0.1-219) (n=41) | 0.65 |
| **Log NT-proBNP (pmol/ml)*** | 8.4± 1.3(n=29) | 8.0±1.0(n=40) | **0.037** |
| Total Cholesterol (mg/dl) Median(range) | 224(7.8-352) (n=15) | 203.5 (92-451) (n=22) | 0.54 |
| CK (U/l) Median(range) | 62 (0.9-1560) (n=27) | 62(17-1654) (n=36) | 0.93 |

*Data presented as mean±SD

HT: Hypertensive conditions during pregnancy such as pregnancy-induced hypertension, preeclampsia and/or HELLP. Number of patient evaluated is provided behind each value. P-value compared patients with HT vs patients without HT during pregnancy.

**Supplementary Table 4: Baseline characteristics in PPCM patients with and without bromocriptine treatment**

|  | BR-group | Non-BR-group | p-value |
| --- | --- | --- | --- |
| Age (years)* | 33.8 ±6.4 (n=72) | 34.1 ±5.4(n=36) | 0.99 |
| Gravida Median(range) | 2 (1-9) (n=65) | 2 (1-11) (n=28) | 0.68 |
| Parity Median(range) | 2 (1-8) (n=65) | 2 (0-9) (n=30) | 0.98 |
| Caesarean -Section n,(%) | 48/65 (74) | 15/25(60) | 0.20 |
| NYHA n,(%)  I  II  III  IV | 1(2)  11 (19)  22 (37)  25 (42) | 5 (15)  11 (32)  8 (53) | 0.82 |
| Smoking n,(%) | 24/50 (48) | 8/21(38) | 0.60 |
| Systolic blood pressure (mmHg)* | 115 ±19(n=33) | 120 ±24(n=23) | 0.62 |
| Diastolic blood pressure (mmHg)* | 77 ±15(n=32) | 77 ±19(n=24) | 0.78 |
| Heart rate (bpm)* | 98±22 (n=39) | 93 ±30(n=28) | 0.36 |
| LVEDD (mm)* | 60.3 ±7.2(n=48) | 62.3 ±10.1(n=27) | 0.42 |
| LVESD (mm)* | 51.8 ±8.9(n=40) | 51.9 ±10.1(n=27) | 0.65 |
| LVEF (%)* | 27 ±9(n=73) | 26 ±10(n=36) | 0.43 |
| ADMA (μmol/l) Median(range) | 0.69(0.36-1.70) (n=32) | 0.63(0.45-1.64) (n=9) | 0.88 |
| Prolactin (ng/ml) Median(range) | 16.5 (070-213.7) (n=38) | 25.6 (4.7-258) (n=12) | 0.42 |
| Cathepsin D (% activity of control) Median(range) | 186 (72-388) (n=32) | 152 (105-821) (n=11) | 0.48 |
| CRP (mg/l) Median(range) | 9.8 (0.5-219) (n=50) | 8.5 (0.1-147) (n=22) | 1.00 |
| Log NT-proBNP (pmol/ml) | 8.3 ±0.9(n=53) | 7.6 ±1.7(n=16) | 0.03 |
| Total cholesterol (mg/dl) Median(range) | 212 (718-352) (n=23) | 200.5 (92-451) (n=14) | 0.37 |
| TnT (µg/l) Median(range) | 0.02 (0.01-70) (n=35) | 0.01(0.01-38.00) (n=14) | 0.18 |
| CK (U/l) Median(range) | 64 (0.9-1654) (n=43) | 54.5 (10-1560) (n=20) | 0.37 |
| AST (U/l) Median(range) | 29 (12-263) (n=51) | 36 (17-197) (n=20) | 0.37 |
| GGT (U/l) Median(range) | 30.5(4-603) (n=40) | 19 (7-157) (n=15) | 0.15 |
| Hb (g/dl)* | 11.4 ±1.8(n=55) | 12.0 ±2.0(n=21) | 0.43 |

***** Data presented as mean±SD. Cathepsin D activity has been calculated in % of the mean of healthy postpartum controls. P-value compared BR-group vs non-BR-group.

**
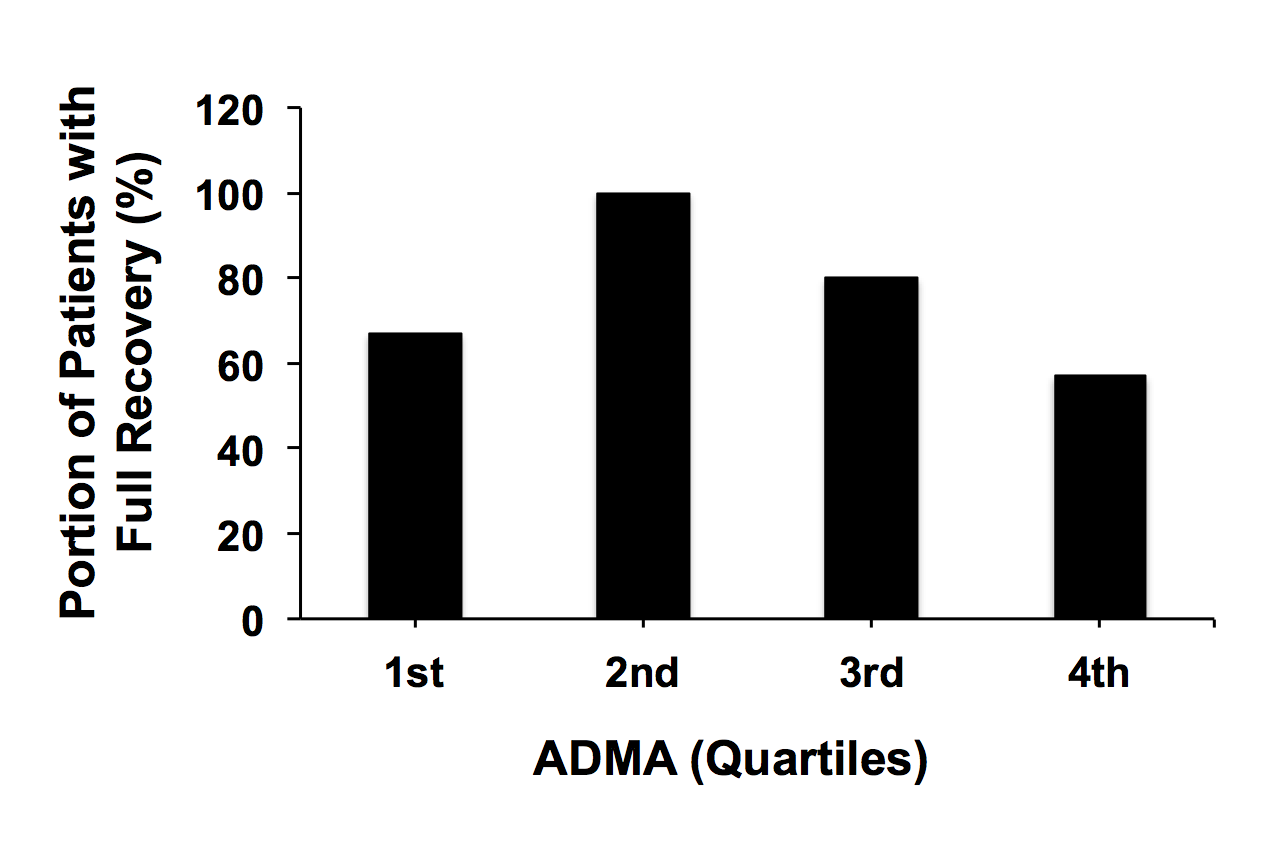
**

**Supplementary Figure 1:** Bar graph depicting the portion of the patients of each quartile with a full cardiac recovery (cut-off values: 1st Quartile: 0.52 µmol/l, n=7; 2nd Quartile: 0.59 µmol/l, n=2, 3rd Quartile: 0.69 µmol/l, n=5; 4th Quartile: 0.85 µmol/l, n=7). No significant differences were observed between groups.
